# Vaccines against Botulism

**DOI:** 10.3390/toxins9090268

**Published:** 2017-09-02

**Authors:** Grace Sundeen, Joseph T. Barbieri

**Affiliations:** Department of Microbiology and Immunology, Medical College of Wisconsin, Milwaukee, WI 53226, USA; gsundeen@mcw.edu

**Keywords:** botulism, botulinum neurotoxins, vaccines, plasmid vectors, viral vectors, toxoids, genetically inactivated toxoids

## Abstract

Botulinum neurotoxins (BoNT) cause the flaccid paralysis of botulism by inhibiting the release of acetylcholine from motor neurons. There are seven serotypes of BoNT (A-G), with limited therapies, and no FDA approved vaccine for botulism. An investigational formalin-inactivated penta-serotype-BoNT/A-E toxoid vaccine was used to vaccinate people who are at high risk of contracting botulism. However, this formalin-inactivated penta-serotype-BoNT/A-E toxoid vaccine was losing potency and was discontinued. This article reviews the different vaccines being developed to replace the discontinued toxoid vaccine. These vaccines include DNA-based, viral vector-based, and recombinant protein-based vaccines. DNA-based vaccines include plasmids or viral vectors containing the gene encoding one of the BoNT heavy chain receptor binding domains (HC). Viral vectors reviewed are adenovirus, influenza virus, rabies virus, Semliki Forest virus, and Venezuelan Equine Encephalitis virus. Among the potential recombinant protein vaccines reviewed are HC, light chain-heavy chain translocation domain, and chemically or genetically inactivated holotoxin.

The CDC recognizes five forms of botulism [1]: **Foodborne botulism**, intoxication occurs upon eating foods contaminated with Botulinum neurotoxins. Improperly prepared homemade foods are a common source of this intoxication; **Wound botulism**, infection occurs upon contamination of a wound by *Clostridium botulinum* spores, which germinate and produce botulinum neurotoxin (BoNT). At risk for wound botulism are intravenous drug users, people who suffered a traumatic injury, or surgery patients; **Infant botulism and Adult intestinal toxemia botulism**, infection follows the ingestion of *C. botulinum* spores. *C. baratii* and *C. butyricum* and other clostridia species cause botulism as well. Spores enter the intestine, germinate, and produce BoNT; and **Iatrogenic botulism**, intoxication occurs when humans overdose from a BoNT injection for cosmetic or medical applications.

**BoNT Structure-function** BoNTs are the most toxic protein toxins for humans [2]. There are seven BoNT serotypes, termed (A-G) with subsequent recognition of natural variants within each serotype termed subtypes [3]. BoNTs are 150-kDa single chain **AB** proteins (Figure 1) cleaved to form a 50-kDa light chain (L) and a 100-kDa heavy chain (H), which are linked by a disulfide bond. Ls are zinc proteases which cleave plasma membrane or vesicle associated Soluble *N*-ethylmaleimide-sensitive factor activating protein receptor (SNARE) proteins, based upon the serotype [4]. H is organized into an N-terminal Translocation domain (HN) and a C-terminal Receptor Binding domain (HC). BoNT neurotoxicity is due to two specific actions; BoNT-L cleaves neuron-specific SNARE proteins [5] and BoNT-HC binds neuron-specific receptors [6,7]. Cleavage of SNAREs at the neuro-muscular junction leads to an inhibition of neurotransmitter release, resulting in flaccid paralysis. Once inside a neuron, BoNT-L can persist in an active form for up to several months, depending on the BoNT serotype [8].

The seven immunologically distinct BoNT serotypes (A–G) [9] are defined when antiserum to one serotype only neutralizes the homologous serotype. In general, BoNT serotypes A, B, E, and F are associated with human botulism and BoNT serotypes B, C, and D are associated with animal botulism [1]. Recent BoNT isolates have been proposed to comprise new serotypes, including BoNT/H, which was shown to represent the chimeric toxin BoNT/FA [10] and BoNT/X, which is not neutralized by antisera to other known BoNT serotypes and cleaves VAMP 2 at a novel site (Arg66–Ala67) [11]. BoNT serotypes share structure-function properties. BoNT serotypes share 40 to 70% primary amino acid sequence homology [12] and possess conserved protein structure-function, including three sequential steps in intoxication: HC (host cell binding); HN (L translocation); and C (catalysis, cleavage of SNARE protein substrates).

Historically, persons at risk have been vaccinated with a formalin-inactivated penta-serotype-BoNT/A–E toxoid as described by Graham and Thorp [13], but the current toxoid stock was discontinued for vaccination due to declining potency [14]. Rusnak and Smith provided a detailed review of past vaccines against botulism [15]. This review will update efforts to produce the next generation vaccine against botulism, including DNA- and protein-based vaccines (Table 1).

## 1. Nucleic Acid-Based Vaccines against Botulism

Plasmid- and viral-based vectors are being developed as platforms for vaccines against botulism. Several viral-based platforms have been developed to allow for expression of immunizing doses of BoNT, primarily HC, in several model systems.

### Plasmid-Based Vaccines against Botulism

Clayton and Middlebrook described an early approach for DNA vaccination, using a plasmid based, CMV-expression system. Plasmid-based vaccination protected mice from intraperitoneal challenge by BoNT/A where protection correlated with the generation of antibodies to HC. These early studies conceptually supported the development of DNA vaccination to neutralize toxin action, and the use of HC as a vaccine candidate, based upon ease and safety of production. Plasmids containing the gene(s) encoding BoNT-HCs are attractive vaccine platforms, based upon the ability to produce large quantities of plasmid. In addition, plasmids are stable, allowing for distribution with limited constraints on storage conditions. Plasmid-based HC expression is enhanced with codon optimization for the host vaccinated [16,17,18,19,20,21]. A gene encoding a signal peptide was added to the N terminus of HC to promote HC secretion and enhance immune response [16,18,19,20]. Jathoul et al. found the human ubiquitin C (UbC) promoter elicited high expression of HC/F and subsequently high HC/F antibody titers, which protected against a challenge by 10,000 MLD_50_ Units of BoNT/F [17] Plasmid-based vaccination may require multiple vaccinations to elicit protective antibody titers [20].

## 2. Viral Vector Vaccines

### 2.1. Adenovirus-Based Vectors

Adenoviruses have linear, double-stranded DNA genomes of 36–38 kb. There are numerous human adenoviruses [22] serotypes, and a replication-incompetent human serotype 5 (AdHu5) has been developed as a viral vaccine vector. AdHu5 vectors lacking E1, and encoding a human codon-optimized gene for BoNT/C HC (Ad/opt-BoNT/C-H_C_50) [23,24,25] has been used to immunize mice via intramuscular injection (2 × 10^7^ pfu). Vaccinated mice surviving challenge with 100 MLD_50_ Units of BoNT/A at seven weeks post-vaccination [25]. Similar challenge experiments were performed with intranasal- [23] or oral- [24] vaccination with similar protection from BoNT challenge. Of note, intranasal vaccination yielded an IgA response, in addition to an IgG response, to the vaccination. Zeng et al. [25] addressed the concern for preexisting adenovirus immunity by showing mice previously vaccinated with AdHu5 followed by vaccination with Ad/opt-BoNT/C-H_C_50 survived challenge with BoNT/C and despite having immunity to AdHu5.

### 2.2. Influenza Virus-Based Vectors

Influenza virus is a negative sense segmented RNA virus which does not replicate through a DNA intermediate. This eliminates the possibility of integrating the viral genome into the host genome; increasing vaccine safety relative to DNA viral vectors. Li et al. [26] developed a live attenuated influenza viral vector as a platform for intranasal vaccination of the C-terminal subdomain of the receptor binding domain of BoNT/A, HCc/A, as a candidate botulinum vaccine. BALB/c mice vaccinated intranasally with 5 pfu followed with a boost at 4 weeks had an IgG response to the viral vector strain (PR8) and BoNT/A and survived challenge by 10 MLD_50_ Units of BoNT/A or 100 LD_50_ doses of WT PR8.

### 2.3. Rabies Virus-Based Vectors

Rabies virus (RABV) has a single-stranded, negative sense RNA genome. A recombinant RABV based on the SAD-B19 strain was used to make candidate vaccines against BoNT [27,28]. DNA encoding HC/A, HC/B, or HC/E were fused to the nucleic acids encoding RABV glycoprotein, viral particles were produced, and HC expression was shown by FACS and Western blots [27,28]. Unexpectedly, attempts to codon optimize HC/E gene were complicated due to the observation that the codon optimized HC/E gene developed a silencing mutation during viral replication, which was not observed for either HC/A- or HC/B- gene. Mice were vaccinated with β-Propiolactone inactivated individual or trivalent vaccines and were given boosters at two and four weeks post primary vaccination [28]. Vaccination with the individual HC elicited an antibody response to the respective HC and the RABV glycoprotein [28] and protected against challenge with 1000 LD_50_ Units of BoNT/A and BoNT/B, but did not protect against a challenge with 1000 MLD_50_ Units of BoNT/E. HC/E vaccination extended mouse survival time [28]. Vaccination with the trivalent HC vaccine protected mice from challenge with BoNT/A or BoNT/B, with partial protection to challenge with BoNT/E [28].

### 2.4. Alphaviruses

#### 2.4.1. Semliki Forest Virus (SFV)-Based Plasmid DNA Replicon Vectors

Semliki Forest virus (SFV) is a zoonotic, encapsulated, positive single-stranded RNA virus used as both a plasmid-based vaccine, and a viral-based vaccine [29,30,31,32]. HC/A plasmid DNA replicon vector (pSCARSHc) derived from SFV stimulated high HC antibody titers [29,30,31,32]. Yu et al. also generated plasmid DNA replicon SFV vectors containing HC/B, HC/E, HC/F, and HC/TeNT as individual vaccine vectors, which stimulated antibody production against the respective HC. Dual-expression vectors for HC/A and HC/B or HC/E and HC/F were also developed and shown to be as effective as the individual vaccine vectors. The pSCARS vaccines protected vaccinated mice from challenges with 1000 MLD_50_ of BoNT/A, /B, /E, or /F [29,30,31,32].

#### 2.4.2. Semliki Forest Virus-Based Viral Vectors

SFV replicon particles have been engineered with genes encoding HC/A, HC/B, HC/E, HC/F, and HC/TeNT (termed VRP-AHc, -BHc, -EHc, -FHc, and THc respectively) [29,30,32]. Three doses of 5 × 10^6^ particles of VRP-Hc were required to protect mice against a challenge with 1000 MLD_50_ Units of the respective BoNT [32]. Vaccination with combinations of HC vaccines also yielded protection against challenge with 1000 MLD_50_ Units of the homologous BoNT serotype [32]. Subsequent studies have developed tetravalent vaccines, with combinations of the four individual VRPs or the two dual-expressing VRPs [29].

#### 2.4.3. Venezuelan Equine Encephalitis Virus-Based Vectors

Venezuelan Equine Encephalitis virus (VEE) is a mosquito borne virus which has a positive sense single strand RNA genome, an envelope, and a capsid. VEE has been examined as a vaccine vector for BoNT, anthrax, and Marburg virus [33,34]. Lee et al. inserted the gene encoding HC/A downstream of the 26S promotor in the VEE genome, replacing genes which encode for viral structural proteins and rendering the virion replicon particles (HC/A-VRP) replication defective [33]. A HC/A-VRP vaccination regimen with two doses of 10^7^ Units protected mice against challenge by 10,000 MLD_50_ Units of BoNT/A [33] with protection extending for up to one-year post vaccination. Mice vaccinated with HC/A-VRP and replicon particles containing genes for *Bacillus anthracis* mature protective antigen (PA-VRP) and Marburg virus glycoprotein (MBGV-GP-VRP) survived challenge by 1000 MLD_50_ Units of BoNT/A several months post vaccination [34].

**Viral Vector Overview** Each viral-derived vaccine system has unique characteristics which contribute to their observed vaccine potencies and utility as vaccine candidates. The viral vectors discussed are human pathogens so vaccination with these viral vectors may be complicated by preexisting immunity to the vector [35,36,37,38,39,40,41]. In the case of adenovirus, the wide variety of adenovirus serotypes allows for the design of an adenovirus vector which is rarely encountered by humans, adenovirus has a broad host range, a tropism for epithelial cells, and vaccination may result in both IgA and IgG responses, and genes with up to 8 kb can be inserted into the adenoviral genome [42,43]. Using influenza virus as vector for vaccines can become complicated because of preexisting immunity from the seasonal flu vaccine [39]. The influenza vector described above is a live attenuated laboratory strain [7] and other strains of influenza may be more suitable for human vaccination. Using Rabies virus as a potential vector for vaccines was reviewed by Gomme et al. [44]. Briefly, RABV is suitable for a potential vaccine vector, since RABV has a simple genome, which can stably incorporate and express genes which increase the genomes size by 55%. While RABV is a human pathogen, RABV have low seroprevelance and the strain used in vaccine vector studies has already been attenuated by passaging through various cell types and does not infect the CNS. The rabies virus can be further attenuated through genetic mutations or be inactivated by treatment with β-Propiolactone [28,45]. SFV and VEE, both alphaviruses, share many vaccine qualities which were reviewed by Lee et al., Choi et al., and Lundstrom [42,43,46]. Alphaviruses can accept foreign genes of about 5 kb which replace the genes encoding the viral structural proteins resulting in replication deficient replicons [42,43]. Production of the replicons requires a helper plasmid encoding the viral structural proteins co-transfected into host cells along with the desired viral genome.

Another complication of viral vectors is the possibility of integration of the viral genome into the human genome. Adenovirus genomes can adversely integrate into the human genome [47]. In contrast, the RNA genomes of influenza, Rabies, Semliki Forest, and Venezuelan Equine Encephalitis viruses do not use a DNA intermediate which removes the possibility of integration of the vector into the host genome.

## 3. Protein-Based Vaccines against Botulism

Protein-based BoNT vaccines include both native, chemically inactivated toxoids, and recombinant-engineered BoNT vaccines.

### 3.1. Chemically Detoxified BoNT Vaccine

Chemically detoxified BoNT remains a viable approach towards the production of a vaccine stock for personnel at risk. For example, a tetravalent BoNT serotype /A, /B, /E, and /F toxoid vaccine has been engineered derived from progenitor M toxin. Vaccinated human volunteers did not show serious clinical adverse events from toxoid vaccination and antisera collected 1 month after a primary vaccination and three boosts neutralized low amounts of BoNT [48,49,50]. Another study showed nasal immunization of BoNT/A toxoid and a mutated cholera toxin yielded BoNT-specific IgG in plasma and IgA in secretions. Mice receiving this nasal vaccine were protected for challenged with 4000 LD_50_ Units of BoNT/A by IP challenge and by 2 LD_50_ Units of oral delivered progenitor BoNT/A. These studies support the continued development of BoNT vaccines that protect against mucosal BoNT intoxication [48].

### 3.2. Recombinant BoNT Vaccines

HC, LHN, and full-length BoNT have been utilized as candidate vaccines against botulism. These vaccine candidates have been produced in clostridia, *Escherichia coli*, and the yeast, *Pichia pastoris.*

#### 3.2.1. Recombinant, HC-Derived Vaccines

Middlebrook and coworkers engineered and expressed BoNT/A1-HC in *E. coli* as a fusion protein. Mice vaccinated with the fusion protein produced an antibody response to the HC and partially protected mice from challenge by 1200 LD_50_ Units of BoNT/A [51]. These studies showed the feasibility of producing HC as a protein-derived vaccine candidate and encouraged the continued development of the vaccine potential of HC in several heterologous expression systems [52,53,54,55]. Only a few examples towards developing HC as a vaccine will be described and interested readers are encouraged search for other studies that utilize HCs for vaccine development.

Smith and coworkers utilize the yeast *P. pastoris* to express HC. Mice and non-human primates vaccinated with HC were protected against challenge with the homologous serotype of BoNT and these studies have been extended towards developing a bivalent HC vaccine (termed rBV A/B). Antibodies from serum of clinical volunteers vaccinated with rBV A/B was protective in a guinea pig passive transfer model and a mouse bioassay, showing the neutralizing capacity of rBV A/B. rBV A/B is currently in clinical trials [56]. Expression of the HC of the seven BoNT serotypes (hepta-HC) has been achieved in *E. coli*. Outbred mice vaccinated with hepta-HC elicited an antibody response to each of the seven BoNT HCs and were protected from challenge by 10,000 LD_50_ Units of each of the seven BoNT serotypes. The basis for the neutralization correlated with the ability of HC antisera to block HC binding to gangliosides, the first step in BoNT intoxication of neurons. Other studies showed that mutation of HC to lack ganglioside binding retained the ability to stimulate protective immunity in outbred mice [52,53,54,55]. Thus, there may be several modifications to the HC that may enhance vaccine potency.

#### 3.2.2. Recombinant, LHN-Derived Vaccine

LHN/A, which comprises the catalytic- and translocation-domains, was engineered as a BoNT vaccine candidate [57]. LHN/A was a potent vaccine against BoNT/A challenge. LHN vaccination was effective against homologous and heterologous BoNT subtypes challenge and showed single-dose protection against the principal toxin subtypes of BoNT/A. The high potency of the LHN vaccine highlights the presence of BoNT neutralizing epitopes within L and HN, independent of HC.

#### 3.2.3. Engineered, Full-Length BoNT Vaccines

Full-length BoNT has been engineered to possess reduced catalytic activity and toxicity by mutating residues involved in Zinc binding [58,59] within L in clostridia [60], *E. coli* [61], and *P. pastoris* [62]. In each case, multiple amino acids were mutated involved in coordinating Zn^++^ to reduce catalytic potential. Feasibility for the development of the full-length BoNT derivatives as immunogens is provided by the determination that the structure of full-length BoNT/A1 with a 3-amino acid mutation (E224A/R363A/Y366F) was similar to native BoNT/A1 [63].

## 4. Veterinary BoNT Vaccines

An international workshop on animal botulism was held in 2012 to increase awareness among veterinarians of animal botulism which was underreported and underdiagnosed [64]. The workshop identified several areas for the study of animal botulism, including an increase in the understanding of the botulism in animals, developing new vaccines and diagnostics, and organizing the European community to increase surveillance.

Botulism in livestock is often a food-borne intoxication caused by the ingestion of preformed BoNT serotypes B, C, or D [65,66,67,68,69]. Vaccine efforts in veterinary botulism is more restricted relative to human botulism, and includes chemically-inactivated toxoid and recombinant HC. Vaccines include chemically-inactivated toxoids of BoNT/B, BoNT/C, and BoNT/D [53]. Current preventative efforts address optimizing vaccination schedules [70] and determining the feasibility of developing HCs to increase vaccine coverage with limited reactivity [71].

## 5. Summary

Strategies for the development of vaccines against botulism utilize two approaches; using a native BoNT to generate chemically-inactivated toxoid, or using recombinant techniques to engineer BoNT derivatives. Based upon the ability to produce large quantities with stability in storage, plasmid-based vaccines are attractive, but may require multiple boosts and modified expression strategies to optimize potency. Each viral-derived vaccine system has unique characteristics which contribute to their observed vaccine potencies and utility as vaccine candidates, but vaccination may be complicated by preexisting immunity. Chemically-inactivated native BoNTs are proven vaccine platforms which could be modified to optimize potency. Of the recombinant BoNT-derivatives under investigation, HC-derived vaccines are safe to produce and are protective in vaccine challenges, but appear less potent relative to the multi-domain LHN and full-length BoNT derivatives. However, the potential for multi-domain BoNT vaccines to revert back to native activity must be considered [72]. Ongoing studies towards developing the next generation BoNT vaccine will influence the next generation of vaccines against other bacterial toxins and virulence factors, as new vaccine candidates are continually being defined through reverse vaccinology [73] and informatics [74,75], among other approaches.

## Figures and Tables

**Figure 1:**
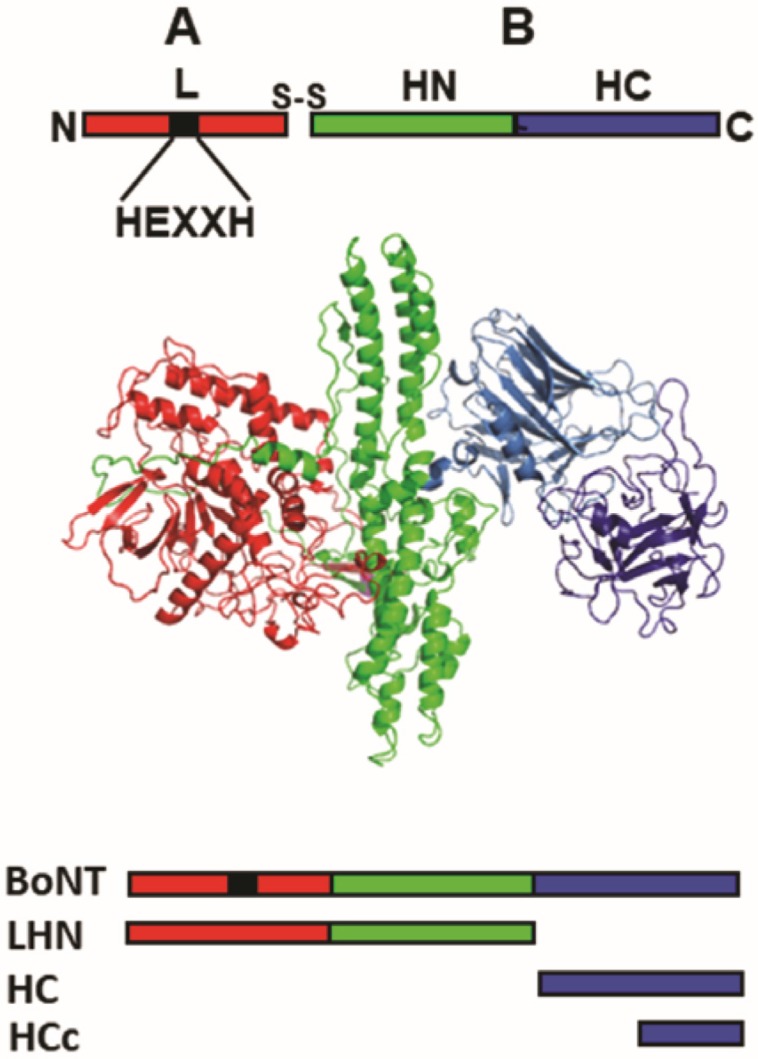
**BoNT Structure-Function**. (**Upper**) BoNTs are 150-kDa single chain proteins cleaved by bacterial or host proteases to a 50-kDa light chain (L, red) and a 100-kDa heavy chain (H), which are linked by a disulfide bond. H is organized into an N-terminal Translocation domain (HN, green) and a C-terminal Receptor Binding domain (HC, blue). L is a zinc metalloprotease with a conserved HEXXH motif (^▄^) that coordinates the metal and is often subjected to mutagenesis to reduce catalytic activity (cytotoxicity) for multidomain vaccine candidates and structure-function studies. (**Middle**) Crystal structure of BoNT/A (PDB:3BTA). Note the independent nature of the three functional domains. (**Lower**) Organization of the various BoNT-derivatives which have been used in recombinant DNA-based and protein-based vaccines listed top to bottom; BoNT, LHN, HC, and HCc.

**Table 1:** Vaccines against botulism.

Material	Vaccine Vector		Vaccine Properties		Comments
Nucleic acid	Plasmid		HC-based vaccine		Simple production, storage, and distribution Multiple vaccinations to elicit protective antibody titer
	Plasmid DNA replicon		Semliki Forest Virus genome with HC replacing the structural proteins		Simple production, storage, and distribution Bivalent and tetravalent vaccines Multiple vaccinations elicited protective antibody titer
Viral Particle	Virus		Genome	Attenuated/Inactivation	
	Adenovirus		Linear dsDNA	Inactivated—lacks the E1 gene	Intranasal vaccine produced IgA Numerous serotypes Preexisting immunity Viral genome may integrate into host genome Potent
	Influenza		Segmented -ssRNA	Attenuated	Multiple vaccinations elicited protective titer to HC/Influenza Preexisting immunity Seasonal influenza vaccine may interfere with HC potency Viral genome unlikely to integrate into host genome Least potent
	Rabies		-ssRNA	Attenuated and inactivated by β-Propiolactone	Multiple vaccinations elicited protective antibody titer Vaccination yielded antibodies to HC/Rabies glycoprotein Low seroprevalance Viral genome unlikely to integrate into host genome Potent
	Alphaviruses	Semliki Forest	+ssRNA	Inactivated—lacks the genes for structural proteins	Bivalent and tetravalent vaccines are possible Multiple vaccinations to elicit protective antibody titer Preexisting immunity Viral genome is unlikely to integrate into host genome Potent
		Venezuelan Equine Encephalitis			Multiple vaccinations elicited protective antibody titer Preexisting immunity Viral genome unlikely to integrate into host genome Potent
Protein	Toxoid		Chemical inactivation		Preparation may reduce immunogenicity Complex production Potent
	Recombinant HC		Receptor binding domain		Simple Production, maybe be useful for rapid BoNT neutralization Least Potent
	Recombinant LHN		Light chain and Translocation domain		Missing neutralizing HC epitopes Potent
	Recombinant BoNT		Light chain and Heavy chain		Possesses all neutralizing epitopes Potent
